# On the Doses of Active Medicines

**Published:** 1807-07

**Authors:** 


### *To the Editors of the Medical and Phyſical Journal.*

Gentlemen,

As every attempt, however incompetent the author may be, to add a mite to the improvement of Medicine, in any of its branches, must be received by you with your usual candour and liberality, I shall make no farther apology in offering the following remarks. If they appear to you consistent, and meet with your approbation, the insertion of them will be deemed a favour.

Medicine, in all its branches, has received considerable improvements and discoveries within these late years, from the indefatigable researches of learned and scientific men; but still, with respect to the practice of medicine, there are diseases that altogether baffle the utmost stretch of the healing art, and for which no adequate remedies have yet been discovered. It has been suggested by some that there is a remedy (though latent and concealed) for every malady incident to mankind; but this is a mere supposition. However, in my opinion, there are many powerful and active medicines of the materia medica, which, if given in larger doses than ordinary, might be of infinite service in obstinate disorders that will not in the least yield to remedies of a milder class. Inveterate and fatal diseases require quick and powerful medicines; and it must be recollected that active medicines have a very different effect on the constitution in a state of health than that of disease. For instance, cantharides even taken to the extent of a few grains, have been known in a state of health, to produce the most dreadful effects; bloody urine, purulent and mucous stools, faintings, spasms, &c.; whereas, in a state of disease, when the viscera are overloaded, and the kidneys and ureters obstructed with thick viscid mucous matter, cantharides given in very considerable doses, *have* been known to have excellent effects. This evidently demonstrates, that the mucous matter that lined (as it were) the ureters, &c. defended the parts from the extreme acrimony of the fly. I am the more confirmed in my opinion, that much benefit might be derived from the exhibition of larger doses of these powerful drugs in inveterate and alarming disorders, from the happy termination of the most distressing complaints, through the inadvertency of nurses in giving external applications for internal medicines. Such mistakes, though no excuse for the negligence of the attendants, have been the means of restoring the patient to health and vigour. Among many other cases, none can be more in point than *that* related in your last, which evidently shows, that what a medical practitioner would shudder at giving as an internal remedy, in such large doses, was given with no detriment, but, on the contrary, with unexampled success.

I would not, however, by these observations, wish that private practitioners should make their patients the test of rash experiments; this would be unjustifiable in the highest degree: but in large hospitals, where there are a number of patients lying under the most distressing and fatal diseases, would it not be expedient to try the effect of increased doses of the more powerful and efficacious drugs, in different diseases, in order that they may be afterwards administered with safety and certainty in general practice. The only desire I have in suggesting these remarks is the good of mankind; for as medicines, when skilfully administered, are the only means of counteracting and resisting the ravages of disease, we ought to investigate to the utmost in our power, the nature and specific virtues of active and efficacious drugs.

